# Modeling Pain Dynamics and Opioid Response in Oncology Inpatients: A Retrospective Study with Application to AI-Guided Analgesic Strategies in Colorectal Cancer

**DOI:** 10.3390/medicina61101741

**Published:** 2025-09-25

**Authors:** Eliza-Maria Froicu (Armeanu), Oriana-Maria Onicescu (Oniciuc), Ioana Creangă-Murariu, Camelia Dascălu, Bogdan Gafton, Vlad-Adrian Afrăsânie, Teodora Alexa-Stratulat, Mihai-Vasile Marinca, Diana-Maria Pușcașu, Lucian Miron, Gema Bacoanu, Irina Afrăsânie, Vladimir Poroch

**Affiliations:** 1 Department of Oncology, Faculty of Medicine, “Grigore T. Popa” University of Medicine and Pharmacy, 700115 Iasi, Romaniagaftonbogdan@yahoo.com (B.G.); teodora.alexa-stratulat@umfiasi.ro (T.A.-S.); m.marinca@gmail.com (M.-V.M.); tanasamdiana@gmail.com (D.-M.P.); lucmir@gmail.com (L.M.); 2 2nd Internal Medicine Department, Faculty of Medicine, “Grigore T. Popa” University of Medicine and Pharmacy of Iasi, 700115 Iași, Romania; gema.bacoanu@umfiasi.ro (G.B.); vladimir.poroch@umfiasi.ro (V.P.); 3 Faculty of Computer Science, “Alexandru Ioan Cuza” University, 700506 Iasi, Romania; oriana.oniciuc@info.uaic.ro; 4 Department of Medical Oncology, Regional Institute of Oncology, 700483 Iasi, Romania; 5 Advanced Center for Research and Development in Experimental Medicine “Prof. Ostin C. Mungiu”, “Grigore T. Popa” University of Medicine and Pharmacy, 700115 Iasi, Romania; camelia-dnascut@email.umfiasi.ro; 6 Centre for Translational Medicine, Semmelweis University, 1085 Budapest, Hungary; 7 Department of Pharmacology, Clinical Pharmacology and Algesiology, “Grigore T. Popa” University of Medicine and Pharmacy, 16 Universitatii Street, 700115 Iasi, Romania; 8 Department of Medical Genetics, Faculty of Medicine, ‘Grigore T. Popa’ University of Medicine and Pharmacy, 700115 Iasi, Romania; 9 Department of Palliative Care, Regional Institute of Oncology, 700483 Iasi, Romania; 10 Department of Internal Medicine, Faculty of Medicine, “Grigore T. Popa” University of Medicine and Pharmacy, 700115 Iasi, Romania; irina.afrasanie@umfiasi.ro

**Keywords:** cancer pain, colorectal cancer, analgesic ladder, artificial intelligence, pain models

## Abstract

*Background and Objectives:* Cancer pain continues to be a major clinical problem nowadays. This study aims to evaluate the World Health Organization (WHO) analgesic ladder effectiveness in patients with colorectal cancer and develop machine learning models to predict treatment response for precision pain management. *Materials and Methods:* In a retrospective observational study, a total of 107 oncological patients were analyzed, with a detailed subgroup analysis of 42 patients with colorectal cancer, hospitalized between July and September in 2022. The pain assessment used numerical rating scales at baseline and 2–3 weeks follow-up. Clinical variables included demographics, disease staging, metastatic patterns, analgesic progression, and medication usage. Machine learning algorithms (e.g., Random Forest, CatBoost, XGBoost, and Neural Network) were used to predict pain reduction outcomes. The UMAP dimensionality reduction and clustering identified the patient phenotypes. *Results:* Statistical analyses included descriptive methods, Chi-square and Mann–Whitney tests, and the models’ performance was evaluated by AUC. Among patients with colorectal cancer, 73.8% achieved clinically pain improvement, with a mean reduction of 2.62 points and median improvement of 3.00 points. The metastatic site significantly affected outcomes: visceral metastases patients showed median improvement of 3.00 points with high variability, patients with bone metastases demonstrated heterogeneous responses (range: −2.00 to +8.00 points), while non-metastatic patients exhibited consistent improvement. Random Forest achieved optimal predictive performance (AUC: 0.9167), identifying the baseline pain score, bone metastases, Fentanyl usage, anticonvulsants, and antispasmodics as key predictive features. The clustering analysis revealed two distinct phenotypes, requiring different analgesic intensities. *Conclusions:* This study validates the WHO analgesic ladder effectiveness while demonstrating superior outcomes in patients with colorectal cancer. The machine learning models successfully predict the treatment response with excellent discriminative ability, supporting precision medicine implementation in cancer pain management.

## 1. Introduction

Cancer pain remains a significant clinical challenge despite therapeutic advances, with pain prevalence closely linked to disease progression through tumor growth, metastatic spread, and tissue invasion. In the research conducted by Beuken-van Everdingen et al. (2016), it was highlighted that the incidence of pain intensifies with disease advancement, affecting approximately 64% of the patients with terminal-stage cancer [1], with a profound impact on quality of life. This explains the reason why the effective pain management strategies are essential in the oncology care.

The analgesic ladder, proposed by the WHO, was published in 1986, which allowed the standardization of the analgesic therapy according to the pain intensity [2]. The application of the principles proposed by the analgesic ladder showed that it can control pain in 70–90% of the cancer patients [3]. Ferreira and colleagues (2006) discussed in their work that a complete elimination of pain is rarely achieved in the cancer patients, but its intensity or duration can be reduced through the use of the WHO guidelines [4]. The WHO analgesic ladder recommends a stepwise approach, including non-opioid medications for mild pain, weak opioids for moderate pain, and strong opioids for severe pain. Adjuvant medications are added at any step based on the specific type of pain (neuropathic, visceral, or bone pain). Despite extensive evidence regarding cancer pain prevalence and the WHO analgesic ladder’s established efficacy, recent trends are reconsidering this methodology for pain management based exclusively on intensity [5,6].

The primary objective of our study is to understand which patient characteristics predict treatment response and establish their clinical utility in the WHO ladder-guided analgesia. Rather than focusing on validating or questioning this approach, our interest lies in characterizing real-world pain trajectories and identifying responders subgroups by using advanced analytical techniques [7]. By including a clustering analysis, we identify distinct patient subgroups based on pain severity and treatment responses. A particular focus is placed on examining correlations between specific analgesic medications and the degree of pain reduction achieved, allowing for evidence-based evaluation of treatment efficacy according to the WHO analgesic ladder principles.

Therefore, our study investigates pain evolution and opioid use between two consecutive inpatient visits in a cohort of oncology patients. By applying machine learning techniques such as clustering, we aim to uncover pain profiles based on tumor characteristics, presence of metastases, opioid dosing patterns, and response to treatment. The colorectal cancer was selected as our primary focus due to its distinctive metastatic behavior, characterized by predictable progression to liver and lung involvement, which creates well-defined visceral pain patterns amenable to a systematic analysis.

## 2. Materials and Methods

### 2.1. Study Characteristics

An observational, retrospective study was conducted to analyze physical pain and its management in 107 patients hospitalized during July–September 2022 in the inpatient oncology ward at the Regional Institute of Oncology Iași. The retrospective design enabled the collection and analysis of data from patients’ medical records.

### 2.2. Population Characteristics

**Figure 1:**
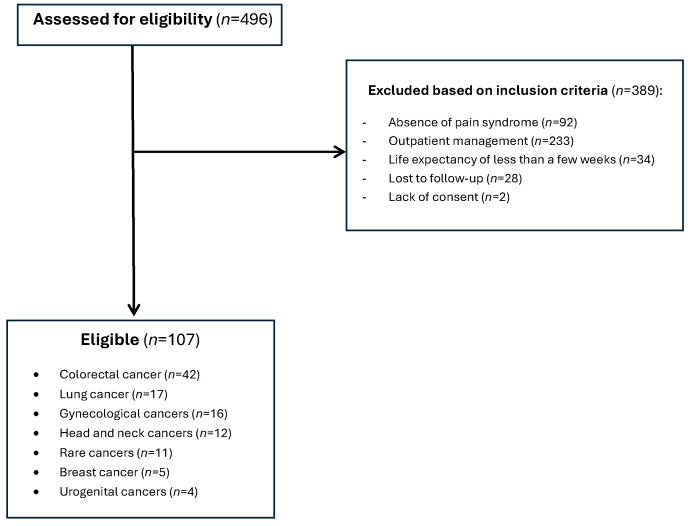
Patient selection flowchart.

The study population included patients with neoplastic pathology requiring active oncological treatment (cytotoxic chemotherapy, targeted molecular therapy, immunotherapy, hormone therapy), hospitalized in the inpatient Oncology unit at the Regional Institute of Oncology Iași. We screened 496 patients receiving active oncological treatment in the mentioned timeframe, among them, 107 met the inclusion criteria and were included in the analysis (Figure 1).

### 2.3. Inclusion and Exclusion Criteria

The inclusion criteria for the study comprised patients aged over 18 years with oncological disease confirmed by histopathological examination, presence of pain syndrome Numerical Pain Scale (NPRS) > 4, associated analgesic treatment in the therapeutic regimen, hospitalization in inpatient regime, and presentation for two consecutive visits. The exclusion criteria included patients with undiagnosed oncological disease, negative prognosis measured in days or weeks, absence of analgesic medication, unconfirmed pain syndrome, lost to follow-up, and all other patients that did not meet the inclusion criteria. These criteria ensured the selection of adult cancer patients with documented moderate to severe pain, requiring active analgesic management and sufficient follow-up period to assess the treatment response (Figure 1).

The instrument used in the description of pain was the NPRS characterized by numbers from 0 to 10, where 0 represents absence of pain and 10 the most severe pain possible. On this scale, scores up to 4 represent mild pain, 4–6 moderate pain, and values up to 10 are attributed to severe pain.

Pain assessment was conducted at two distinct time points to evaluate the treatment response over the study period. Baseline pain measurements were obtained during the initial patient presentation (Visit 1), establishing the severity of pain symptoms at the commencement of analgesic treatment. The follow-up pain assessments were performed 2–3 weeks later during the subsequent scheduled visit (Visit 2).

The analgesic medications, used as pharmacological interventions, included non-opioids as nonsteroidal anti-inflammatory drugs (NSAID), metamizole, antispasmodics, and weak and strong opioids, selected according to pain intensity following the WHO pain ladder. These medications were combined with adjuvants, selected according to the pathophysiological type of pain.

### 2.4. Data Collection

A database was built including information about the primary tumor localization, stage, presence of metastases, baseline pain scores, pain treatment type and dosage, type of pain (somatic, visceral, bone, neuropathic, mixed), and evolution of pain intensity between scheduled visits. Data were collected from patients’ medical files and internal treatment registers.

### 2.5. Statistical Methods Used for Data Analysis

The data analysis was performed using standard descriptive statistical methods, such as mean, standard deviation, median, and frequency and proportion for categorical variables. The Chi-square test was used to evaluate correlations between categorical variables.

The significance limit (*p*-value) for rejecting the null hypothesis (H0, no relevant differences exist between analyzed variables) was established a priori at 0.05 for all tests performed in the context of the relatively small number of cases examined. The missing values were noted and excluded from the statistical analyses, except for frequency analyses (for better representation of the study group), to avoid affecting the results of these analyses and maintain the most faithful reproduction of the real situation, in which many of these data are unknown.

Due to the retrospective nature of this real-world data study, an a priori power calculation was not performed as sample size was determined by available patients meeting the inclusion criteria. Post hoc power analysis revealed adequate power for primary analyses: the global cohort (*n* = 107) could detect pain reductions ≥ 1.22 points and the colorectal subgroup (*n* = 42) could detect effects ≥ 1.64 points, both exceeded by observed reductions of 1.78 and 2.62 points, respectively. For binary outcomes, power differed between cohorts with the global sample capable of detecting 26.0% differences between response rates (Cohen’s h = 0.547, 95% CI: 0.153–0.941), while the smaller colorectal subgroup required larger 39.0% differences for adequate detection (Cohen’s h = 0.895, 95% CI: 0.224–1.565). Small metastasis subgroups (*n* = 8 each) were underpowered, only capable of detecting very large effects greater than 5.07 points.

The groups were compared using the Chi-square test, and the quantitative and ordered variables were compared using the Mann–Whitney U test. For comparisons among three or more groups, a one-way ANOVA was used. A two-sided *p*-value ≤ 0.05 was considered statistically significant. Python (version 3.10.9) was used to perform statistical analyses. The following Python packages were listed: ‘pandas’, ‘keras’, ‘sklearn’, ‘seaborn’, and ‘matplotlib’. The demographic differences between the two subgroups were tested using either the Student’s t-test or the Pearson chi-square test.

### 2.6. Dimensionality Reduction and Pattern Recognition

The Uniform Manifold Approximation and Projection (UMAP) dimensionality reduction method was performed on the dataset using 27 clinical features for 107 patients. This analysis enabled visualization and exploration of high-dimensional patient data in a reduced dimensional space to identify potential clustering patterns and relationships between patients based on their comprehensive clinical characteristics.

**Figure 2:**
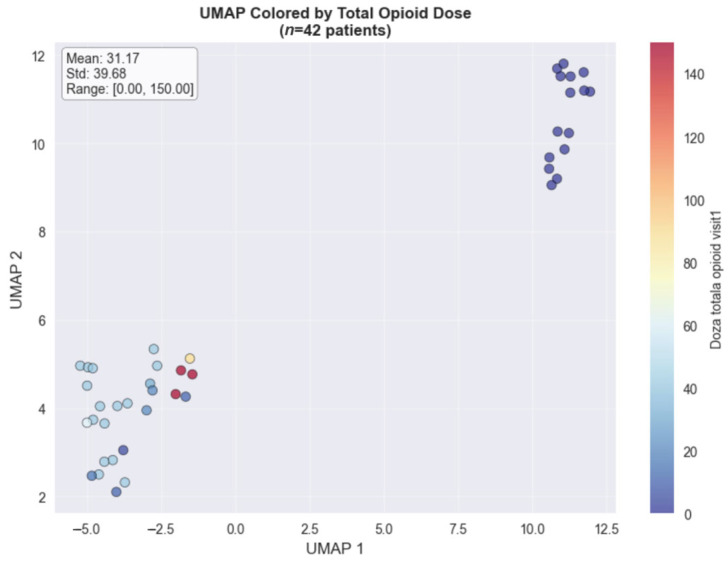
**The** UMAP analysis by total opioid dose.

**Figure 3:**
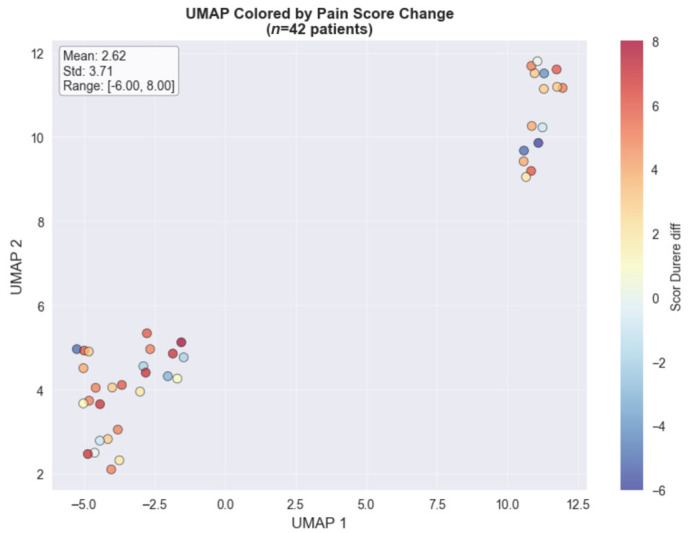
The UMAP analysis by pain score change.

The UMAP dimensionality reduction method, used for visualizing the colorectal cancer subset (*n* = 42), revealed distinct clustering patterns when colored by total opioid dose (Figure 2) and pain score changes (Figure 3). The opioid dose visualization shows clear spatial separation between patients requiring minimal doses (blue cluster, left) and those needing intensive opioid management (red cluster, upper right), with a mean dose of 31.17 mg (range: 0–150 mg). When colored by pain score changes, the high-dose cluster predominantly achieved substantial pain improvements (4.00–8.00 point reductions), while the low-dose cluster showed variable outcomes. This spatial correlation in the colorectal cancer cohort demonstrates that clinical features successfully distinguish between distinct pain management phenotypes based on both treatment intensity and therapeutic response.

### 2.7. Machine Learning Models

We developed categorical machine learning models to predict whether patients would experience pain improvement or deterioration between consecutive visits. The predictive framework used clinical data from the initial visit (Visit 1) as input features to forecast pain score changes at the subsequent visit (Visit 2). The clinical variables were systematically organized into three categories: demographic characteristics (age, gender), disease-related factors (cancer staging, metastatic distribution patterns), and therapeutic interventions (WHO analgesic ladder progression, opioid delivery methods, and specific analgesic medications). This structured approach enabled a comprehensive assessment of how the baseline patient characteristics and initial treatment decisions affect subsequent pain management outcomes.

**Figure 4:**
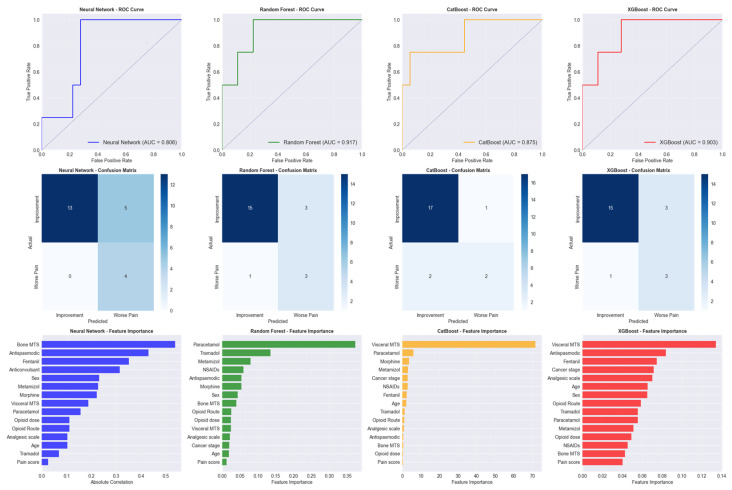
Model performance analysis for pain prediction outcomes.

Four advanced machine learning algorithms were evaluated for predicting pain reduction outcomes: Random Forest, an ensemble method that combines multiple decision trees to improve prediction accuracy and reduce overfitting; CatBoost, a gradient boosting algorithm specifically designed to handle categorical features effectively; XGBoost, an optimized gradient boosting framework known for high performance in predictive modeling tasks; and Neural Network, a deep learning model that mimics brain neural connections to identify complex patterns in data (Figure 4).

The models implemented to predict significant pain reduction used 18 clinical features for 107 patients. The dataset was partitioned into training (*n* = 85) and test (*n* = 22) sets, with notable class imbalance reflecting the clinical reality where pain degrading occurs less frequently (about 25% of the cases).

**Figure 5:**
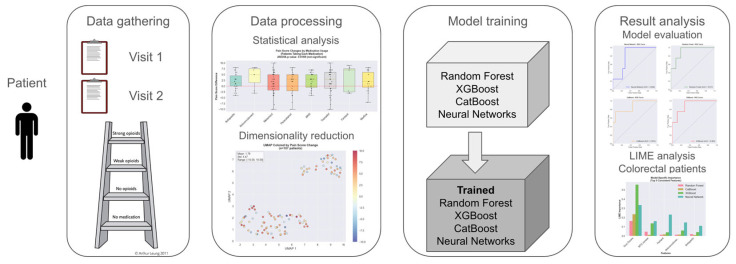
Integrated machine learning framework for cancer pain management.

The Local Interpretable Model-agnostic Explanations (LIME) analysis was conducted across 22 test samples and four machine learning models were used to provide individual-level prediction explanations and identify the most reliable predictive features. The complete machine learning methodology is illustrated in Figure 5, which depicts the four-phase workflow from initial data collection through the final interpretability analysis using LIME (Figure 5).

### 2.8. Ethical Considerations and Necessary Approvals

The study was conducted in accordance with ethical standards and obtained approval from the clinical research ethics committee of the Regional Institute of Oncology Iași, opinion no. 302/26.06.2024. All patient data were anonymized to ensure their confidentiality.

## 3. Results

### 3.1. Patients’ Characteristics

The analysis included data from 107 eligible patients, baseline characteristics are presented in Table 1. In the study group, female patients were more than male patients (77% vs. 23%). The median age in the study group was 63 years, and most patients were 65 years or older (53.8%).

The distribution of primary tumors in our study population (*n* = 107) is formed by colorectal cancer represented by the largest subgroup with 42 patients (39.2%); lung cancers comprised 17 cases (15.9%); and gynecologic malignancies accounted for 16 patients (15.0%). Head and neck cancers were present in 12 patients (11.2%), and rare cancers represented 11 cases (10.3%). Patients with breast cancer were 5 (4.7%), and urogenital malignancies were the least represented with 4 patients (3.7%).

**Figure 6:**
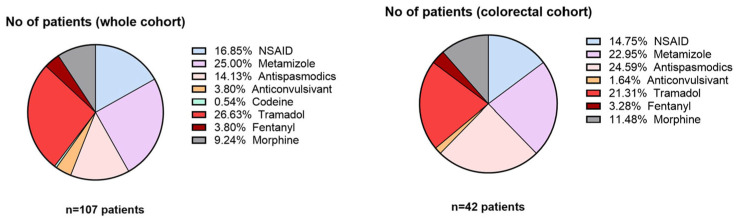
Pain treatment distribution among cohorts.

Between metastatic patients, liver metastases were the most frequent (49.6%), followed by peritoneal metastases (24.6%). Bone and lung metastases were less common, appearing in case of 9.2% and 16.6% of the patients, respectively. Among patients with colorectal cancer, visceral metastases were predominant (26 patients, 61.9%). Bone metastases and non-metastatic disease showed equal distribution, with 8 patients each (19.0%). Regarding treatment type, 40% of patients used some type of opioid treatment (weak or strong), either Tramadol, Codeine, Fentanyl, or Morphine, as presented in Figure 6.

### 3.2. Distribution of Pain Score Characteristics

Pain measurements were systematically recorded at two clinical encounters separated by a 2–3 weeks interval. The initial assessment captured baseline pain intensity at treatment commencement, while the second evaluation documented therapeutic response following implementation of the WHO-guided analgesic protocols.

**Figure 7:**
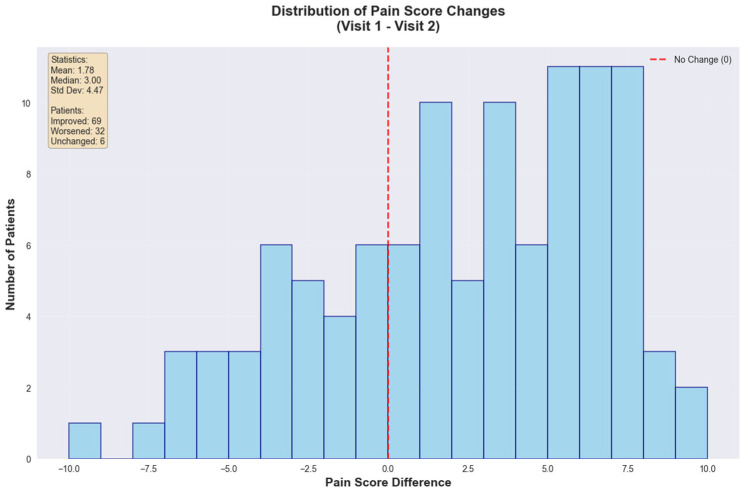
Pain score changes between consecutive visits.

The global statistical analysis revealed a mean pain reduction of 1.78 points (SD = 4.47) with a median improvement of 3.00 points. Patient outcomes showed that 69 patients (64.5%) experienced improvement, 32 patients (29.9%) worsened, and 6 patients (5.6%) remained unchanged, emphasizing that approximately two-thirds (~66%) of the cohort achieved important pain reduction (Figure 7).

**Figure 8:**
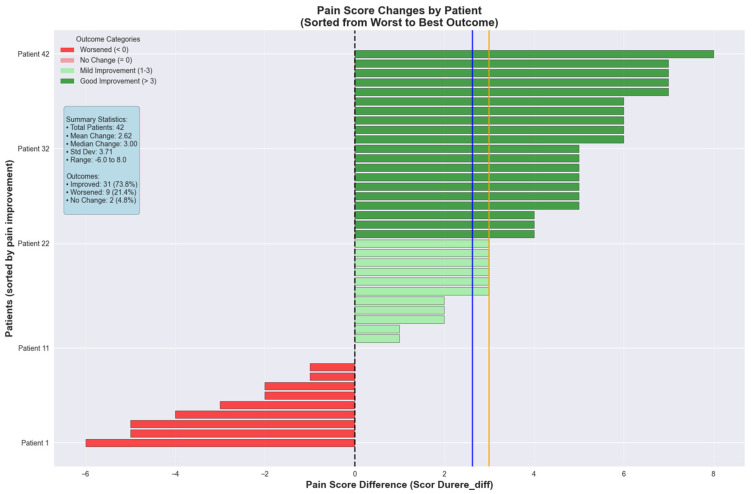
Pain score changes between consecutive visits for colorectal cancer cohort.

The colorectal cancer cohort was further analyzed for pain score changes between consecutive visits. The pain intensity decreased with a mean of 2.62 ± 3.71 points between the two visits, indicating that 50% of the patients experienced a pain reduction of 3.00 points or greater. The range varied from 6.00 points deterioration to 8.00 points improvement. The colorectal cancer cohort demonstrated superior pain management outcomes with a mean reduction of 2.62 ± 3.71 points and median improvement of 3.00 points. Patient outcomes showed 31 patients (73.8%) experienced improvement, 9 patients (21.4%) worsened, and 2 patients (4.8%) remained unchanged (Figure 8).

### 3.3. Impact of Metastatic Site on Pain Management Outcomes in Colorectal Cancer

**Figure 9:**
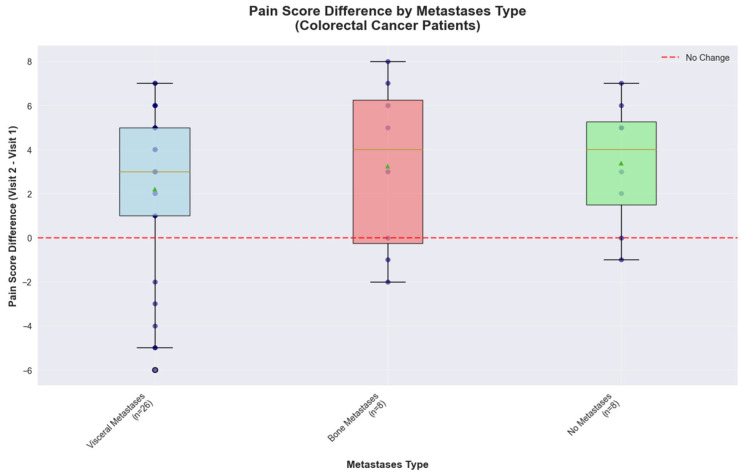
Pain score difference by metastases type in colorectal cancer cohort.

Metastasis localization of colorectal cancer patients influences the pain scores and reveals a distinct response pattern. Patients with visceral metastases (*n* = 26) showed a median pain improvement of 3.00 points, with responses ranging from −6.00 to +7.00 points. Patients with bone metastases (*n* = 8) demonstrated an even better response, with a median improvement of 4.00 points, however they exhibited the greatest variability, with scores ranging from −2.00 to +8.00 points. The high variability observed combined with the small sample size further limits the precision of effect estimates for this subgroup, indicating that larger studies would be needed to definitively characterize metastasis-specific treatment responses. Patients without metastases (*n* = 8) achieved consistent pain reduction with a median improvement of 4.00 points and no cases of pain deterioration, indicating more predictable treatment responses in non-metastatic disease (Figure 9).

These observational findings in a small sample population suggest that colorectal cancer patients may represent a promising target population, with metastasis status potentially serving as a stratification factor for personalized treatment planning and may benefit from targeted investigation in future adequately powered studies.

### 3.4. Class Distribution in Training and Test Sets

The dataset was partitioned into training and test sets with distinct class distributions. In the training step of the machine learning models to recognize patterns and make predictions, patients with no pain decrease represented 67.1% of cases, while those with pain decrease comprised 32.9%. The test step, which evaluates how well the model performs on previously unseen data, showed a more imbalanced distribution, with 81.8% of patients experiencing no pain decrease and 18.2% showing pain decrease. This distribution reflects the inherent class imbalance in pain management outcomes, where significant pain reduction may be less frequent than stable or unchanged pain levels.

### 3.5. Model Performance Comparison

Random Forest emerged as the best-performing model with a test AUC of 0.9167, achieving 81.8% accuracy, 50% precision, and 75% recall. CatBoost achieved the highest test accuracy (86.4%) and precision (66.7%), while Neural Network demonstrated perfect recall (100%) but lower precision (44.4%), and XGBoost showed signs of overfitting with near-perfect training performance but similar test results to Random Forest.

The feature importance analysis revealed that initial pain score was the most predictive factor (importance: 1.000), followed by patient age (0.269), bone metastases presence (0.261), total opioid dose (0.254), NSAID use (0.210), opioid route (0.198), disease stage (0.197), metamizole use (0.195), analgesic step (0.195), and tramadol use (0.190).

### 3.6. 2 LIME Analysis for Model Interpretability

**Figure 10:**
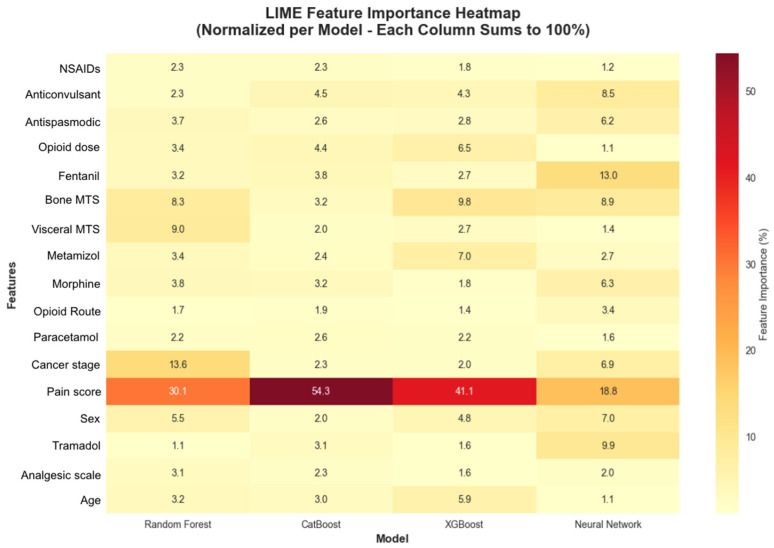
The LIME analysis for model interpretability.

**Figure 11:**
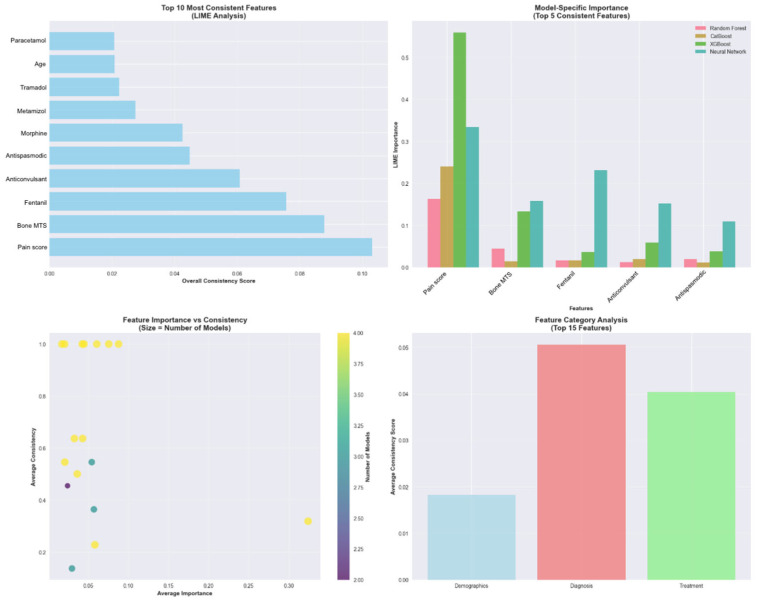
The feature importance analysis for pain prediction models.

The LIME analysis was conducted specifically on the 22 test patients with colorectal cancer, and the results are representative of the clinical particularities and pain management characteristics unique to this type of neoplasia. The analysis identified 17 features with 15 demonstrating statistical significance across different models and patient cases (Figure 10). It revealed that baseline pain score was the most consistently important feature across all models, appearing with 31.8% consistency and the highest consistency score (0.1022). Four additional features demonstrated perfect consistency across all models: bone metastases presence, Fentanyl use, anticonvulsant medication, and antispastic medication, each appearing with 100% consistency across model predictions (Figure 11).

## 4. Discussion

To systematically evaluate predictive factors for pain response in real-world, we implemented a comprehensive machine learning workflow consisting of four sequential phases (Figure 5). The workflow began with structured data gathering from two consecutive patient visits, capturing clinical variables, pain scores, and treatment parameters. Data processing involved statistical analysis and dimensionality reduction techniques to identify key predictive features and visualize patient clustering patterns based on pain score changes. Multiple machine learning algorithms were then trained and compared, including Random Forest, XGBoost, CatBoost, and Neural Networks, with model performance evaluated using standard classification metrics. Finally, result analysis incorporated model evaluation and the LIME analysis specifically for the colorectal cancer cohort to identify the most influential clinical variables driving pain response predictions. This systematic approach enabled both predictive modeling and clinical interpretation of factors associated with treatment success.

Cancer-related pain represents a multifaceted clinical challenge that profoundly affects patient outcomes across diverse oncological populations. Contemporary epidemiological studies demonstrate persistent high prevalence rates despite therapeutic advances, with van den Beuken-van Everdingen et al. 2016 reporting pain prevalence of 50.7% during anticancer treatment, 55.3% during advanced disease, and 66.4% in metastatic settings [8]. Subsequent systematic reviews by Snijders et al. 2023 confirm that pain remains a significant clinical burden, particularly in advanced malignancies where prevalence reaches 44.5% [9]. The clinical significance of effective analgesia extends beyond symptom palliation, where studies found correlations between optimal pain management and improved quality of life, enhanced functional status, and reduced psychological distress in cancer populations [10].

Patient-reported outcome measures have established standardized criteria for treatment success, where clinically important pain reduction being reported as high as ≥30% intensity decrease [11], while others demonstrated that patients consider ≥50% reduction or achievement of pain scores ≤ 3/10 as meaningful therapeutic benefit [12]. The World Health Organization analgesic ladder, first proposed by the WHO Expert Committee 1986, remains the foundational framework for cancer pain management. Comprehensive validation studies by Jadad and Browman (1995) [13] documented significative pain control when protocols were appropriately implemented, while more recent systematic reviews confirm sustained efficacy rates exceeding 50% across diverse populations [2].

Our analysis of colorectal cancer patients showed better results than the established benchmarks, with 73.8% achieving clinically significant improvement compared to the 64–88% success rates reported in mixed cancer populations [14], with our median improvement of 3.00 points surpasses the patient-defined success threshold [12]. These findings align with recent validation studies, demonstrating the WHO ladder effectiveness in contemporary oncological practice [15].

The substantial heterogeneity observed in treatment responses reflects the complex pathophysiology of cancer pain [16]. Individual variation may be attributed to diverse mechanisms including primary nociceptor sensitization, inflammatory mediator release, and central sensitization [17].

A subgroup analysis based on metastatic distribution revealed distinct pain management phenotypes consistent with the literature on site-specific pain mechanisms. Patients with visceral metastases demonstrated variable responses reflecting the complex innervation patterns [18] and the heterogeneous nature of visceral nociception [19]. The pronounced response heterogeneity observed in bone metastases patients aligns previous research [20] documenting the complex pathophysiology involving osteoclast activation, inflammatory cytokine release, and mechanical nociceptor stimulation. Others have specifically characterized the unique therapeutic challenges posed by colorectal cancer bone metastases, where osteolytic processes require multimodal approaches combining opioids, bisphosphonates, and radiation therapy [21].

The machine learning methodologies, used in this study, provided robust validation of clinical observations, with Random Forest performance (AUC: 0.9167) comparable to recent predictive modeling studies in cancer pain [22,23]. The feature importance analysis, confirming the baseline pain severity as the predominant predictor, corroborated the established clinical principles documented by Cleeland et al. 1994 [24] and validated in subsequent studies [25].

The identification of Fentanyl use as a significant predictor reflects its established role in managing severe cancer pain, particularly the visceral pain characteristic of colorectal hepatic metastases. Extensive pharmacological studies have documented Fentanyl’s efficacy in managing continuous, severe pain, while transdermal formulations have provided sustained analgesia for stable pain syndromes [26].

The significance of anticonvulsant and antispasmodic medications as predictive features aligns with growing recognition of neuropathic pain components in cancer [27]. Gabapentin and Pregabalin efficacy in the cancer-related neuropathic pain have been demonstrated in pivotal randomized trials, included as standard of care in oncology guidelines [28]. Moreover, antispasmodic agents address the cramping, colicky pain associated with bowel obstruction [29].

The clustering analysis revealed natural patient stratification into phenotypic groups, supporting personalized medicine approaches in cancer pain populations. This stratification enables precision analgesic selection as proposed by Vollert et al. (2016) [30] and implemented successfully by Cruz-Almeida et al. 2019 [31].

The integration of the WHO principles with machine learning methodologies represents an evolution toward evidence-based precision pain management, proved by neuroimaging studies [32]. These approaches complement emerging opioid stewardship frameworks [33], addressing growing concerns about opioid safety while optimizing therapeutic outcomes. Future research should incorporate respiratory depression prediction models, as developed by Gupta et al. 2018 [34], and pharmacogenomic factors, as characterized by Crews et al. 2014 [35], to achieve comprehensive precision pain management in oncological care.

The integration of the WHO analgesic ladder principles with advanced machine learning techniques allowed for a rigorous, data-driven characterization of pain evolution and facilitated the identification of distinct patient phenotypes, supporting the development of individualized analgesic strategies. The use of standardized NPRS assessments at predefined intervals, combined with dimensionality reduction and interpretability analyses, enhanced both the validity and clinical applicability of the findings. Furthermore, examining a heterogeneous cancer cohort alongside a colorectal cancer subset provided complementary generalizable and disease-specific insights. However, the retrospective, single-center design inherently limits causal inference and generalization, while the modest sample size, particularly in specific metastatic subgroups, may have reduced statistical power. The short follow-up period precluded assessment of long-term analgesic efficacy and opioid-related outcomes, and the reliance on retrospective medical records carries the risk of incomplete documentation. Finally, the absence of patient-reported quality-of-life measures constrains the ability to fully evaluate the broader functional and psychosocial impact of analgesic interventions. These considerations underscore the need for prospective, multicenter studies with extended follow-up and patient-centered outcome measures to validate and expand upon the present findings.

## 5. Conclusions

This comprehensive study demonstrates the continued clinical relevance and effectiveness of the WHO analgesic ladder in managing cancer pain, while advancing the field through innovative machine learning applications. The successful implementation of predictive models offers clinicians evidence-based tools for early identification of patients requiring tailored pain management, optimizing resource allocation while maintaining the systematic approach advocated by WHO guidelines. The colorectal cancer cohort demonstrated superior pain management outcomes compared to the overall population, with most patients achieving meaningful pain reduction. The machine learning methods, with an LIME interpretability, successfully identified key predictive factors for treatment response, while metastasis localization emerged as a significant determinant of pain response patterns. These findings suggest that patients with colorectal cancer represent an optimal target population for this intervention, with metastasis status serving as an important stratification factor for personalized treatment planning. Future research should focus on prospective validation of these predictive models and integration into clinical decision support systems to realize their full potential in improving cancer pain management, also should consider the clear guidance on sample sizes needed for future subgroup analyses.

## Figures and Tables

**Table 1:** Baseline characteristics table.

Characteristics	Total Cohort (*n* = 107)	Colorectal Cohort (*n* = 42)
Age median (range)	63 (55–72)	64 (55–77)
Sex (*n*)	Female *n* = 72 Male *n* = 35	Female *n* = 12 Male *n* = 30
ECOG 0–2 (*n*)	*n* = 98	*n* = 41
ECOG > 2 (*n*)	*n* = 9	*n* = 1
Presence of metastasis (%)	80%	78%
Localization and prevalence of metastasis (%)	Liver 49.6% Peritoneal 24.6% Bone 9.2% Lung 16.6%	Liver 41.9% Peritoneal 29.1% Bone 19% Lung metastasis 10%
Baseline pain score, NRS (range)	1–10	2–8
Non-opioid pain treatment (%)	59.8%	64%
Opioid pain treatment (%)	40.2%	36%

## Data Availability

The data presented in this study are available only upon request from the corresponding author, to protect patients’ privacy.

## Abbreviations

The following abbreviations are used in this manuscript:

AI	Artificial Intelligence
LIME	Local Interpretable Model-agnostic Explanations
NPRS	Numerical Pain Scale
NSAID	Nonsteroidal Anti-Inflammatory Drugs
UMAP	Uniform Manifold Approximation and Projection
WHO	World Health Organization
